# Antibacterial Activity and Chemical Composition of Popular Plant Essential Oils and Their Positive Interactions in Combination

**DOI:** 10.3390/molecules30091864

**Published:** 2025-04-22

**Authors:** Petr Mráz, Marek Kopecký, Lucie Hasoňová, Irena Hoštičková, Alena Vaníčková, Kristýna Perná, Martin Žabka, Marian Hýbl

**Affiliations:** 1 Faculty of Agriculture and Technology, University of South Bohemia in Ceske Budejovice, Studentska 1668, 370 05 Ceske Budejovice, Czech Republic; mrazpe@jcu.cz (P.M.); mkopecky@jcu.cz (M.K.); hasonova@jcu.cz (L.H.); hostickova@jcu.cz (I.H.); kperna@jcu.cz (K.P.); 2 1. Aromaterapeuticka KH a.s., Ksice 11, 349 01 Tachov, Czech Republic; vanickova@aromafauna.eu; 3 Crop Research Institute, Drnovska 507, 161 06 Praha, Czech Republic; zabka@vurv.cz; 4 Institute of Entomology, Biology Centre CAS, Branisovska 31, 370 05 Ceske Budejovice, Czech Republic

**Keywords:** *Staphylococcus aureus*, *Escherichia coli*, minimal inhibition concentration, fractional inhibitory concentration, essential oil/GC-MS/MS

## Abstract

Bacterial diseases are a global problem that threatens human health and cause many deaths each year. The alarming rise in bacterial resistance to modern antimicrobials is particularly concerning. In practice, this necessitates increasing the dosage of antimicrobial agents, posing a potential risk of adverse effects on human health. Additionally, the development of antibiotic resistance is one of the main factors contributing to the ever-growing costs of the global healthcare system. For these reasons, natural and safe antimicrobial agents are increasingly sought after. In this study, the antibacterial activity of 31 different essential oils (EOs) was investigated against *Escherichia coli* and *Staphylococcus aureus*. The most effective EOs were further tested both individually and in dual combinations. Minimum inhibitory concentrations (MICs) and fractional inhibitory concentrations (FICs) were determined to reveal synergistic effects, suggesting potential practical applications. The main bioactive compounds of the EOs with the highest inhibitory activity were identified and quantified using GC-MS/MS analysis. Of the tested EOs, seven demonstrated a strong antimicrobial effect against *E. coli*, most notably oregano (MIC 128 µg/mL) and the thyme/oregano combination (MIC 64 µg/mL, FIC 0.75), while thirteen were effective against *S. aureus*, most notably oregano and garlic (MIC 128 µg/mL),and the pelargonium/garlic combination (MIC 32 µg/mL, FIC 0.375). The pharmaceutical, agricultural, and food industries are promising fields for the application of these safe and natural antimicrobial agents, offering a new range of solutions to combat serious bacterial pathogens.

## 1. Introduction

The global antibiotics market is expected to grow significantly, driven by the rising incidence of infections, the need for new drug development, and increasing awareness of health and hygiene. The growing prevalence of chronic disorders has also heightened the demand for novel treatments. The antibiotics market is projected to generate revenue of USD 58.8 billion by 2027. Current reports predict that the global antibiotics market will grow at a compound annual growth rate (CAGR) of 4% from 2020 to 2027 [1]. The discovery of penicillin expanded the use—and, unfortunately, the misuse—of antimicrobials in human medicine, agriculture, and veterinary applications. This overuse has led to the emergence of resistance, making foodborne diseases a growing global threat [2,3,4]. Furthermore, the rate at which resistance develops far outpaces the development of new synthetic antimicrobials due to the genetic adaptability of pathogens [5]. It is estimated that antimicrobial resistance costs Europe over EUR 9 billion per year and adds USD 20 billion in direct healthcare costs in the United States, in addition to approximately USD 35 billion in lost productivity annually [6].

To address this challenge, researchers have been investigating new antimicrobial agents, with essential oils (EOs) emerging as one of the most promising natural alternatives for controlling bacterial pathogens [7,8,9]. EOs are secondary metabolites derived from plants and contain a variety of biologically active molecules, including terpenoids, phenol-derived aromatic compounds, and aliphatic components. Many of these, particularly the major components (MCs), exhibit significant antimicrobial properties [10]. EOs have been used for medicinal purposes since ancient times due to their antibacterial, antifungal, antiviral, antiparasitic, insecticidal, antioxidant, and antiseptic properties [10,11]. Despite their strong antimicrobial effects, they are considered natural and safe for human consumption (GRAS status) and are environmentally friendly. Their residues are fully biodegradable, posing no hygienic or health risks, making them suitable for use also in organic farming [7,12]. The composition of EOs is crucial, as it directly influences their antimicrobial activity. However, their chemical makeup varies greatly depending on factors such as plant parts, genetic traits, climate conditions, soil properties, and extraction methods. Consequently, there is significant interest in identifying and quantifying the MCs of EOs and evaluating their antimicrobial properties [5,13]. Several studies have demonstrated the strong antimicrobial activity of EOs against foodborne pathogens [7,14,15,16]. This effect is further enhanced by the synergistic interactions of multiple bioactive compounds, which exert antimicrobial action through various mechanisms.

One of the most significant antimicrobial actions of plant oil components is their ability to disrupt microbial membranes. Essential oil compounds interact with the phospholipid bilayer of bacterial cells, increasing membrane permeability and destabilizing the lipid bilayer. For example, thymol and carvacrol have been shown to act synergistically by embedding into the membrane and altering its fluidity, making it more susceptible to osmotic stress and leading to bacterial death [17]. Another study showed that eugenol inhibits the NorA efflux pump in Staphylococcus aureus, allowing antibiotics to accumulate within bacterial cells and thereby increasing their potency [18]. As a result, the development of resistance to EOs is highly unlikely [19]. EOs are typically volatile and have fragrances of varying intensity, which have been utilized in food products as flavoring agents and in human medicine [10,20]. The intensity of an essential oil’s aroma can affect its volatility, which in turn influences its antimicrobial potency. Highly volatile compounds (e.g., menthol and camphor) disperse quickly, resulting in a short-lived but effective antimicrobial action. Less volatile compounds (e.g., eugenol and cinnamaldehyde) persist longer in a system, providing prolonged inhibition of microbial growth [21]. However, their strong scent can sometimes be undesirable. Therefore, the selection of the most potent EOs with the lowest MIC is essential. Using the right combination of EOs is a promising strategy, as it can enhance their antimicrobial activity through synergistic or additive effects [5,22,23].

*Staphylococcus aureus* and *Escherichia coli* are among the most common enteric pathogens in mammals and birds. Certain strains are pathogenic to humans, causing a range of infections, including septicemia, diarrheal diseases, purulent skin infections, nosocomial infections, and extraintestinal conditions such as osteomyelitis, cellulitis, and wound infections [24,25,26]. These bacteria also contribute to food contamination, posing significant threats to food safety and public health [27]. To prevent such contamination and infections, synthetic antimicrobials are widely used. However, growing consumer concerns about the adverse effects of these synthetic agents on human health have driven interest in natural alternatives [28,29,30].

In this study, 31 EOs were evaluated for their antibacterial activity against Gram-positive (*S. aureus*) and Gram-negative (*E. coli*) bacteria, with a focus on EO combinations. Initially, a preliminary screening was conducted using the disc diffusion assay. Subsequently, the compositions of the most effective antibacterial EOs were analyzed, followed by MIC determination using the broth microdilution method. Given that EO combinations or their MCs can exhibit synergistic or additive effects [23], the most potent EOs were tested in all possible dual combinations against both pathogens. The positive interactions of EO combinations, in terms of synergistic or additive effects, were assessed using the fractional inhibitory concentration (FIC) index.

## 2. Results

### 2.1. Screening of EOs for Their Antimicrobial Activity (Agar Disc Diffusion Method)

**Figure 1:**
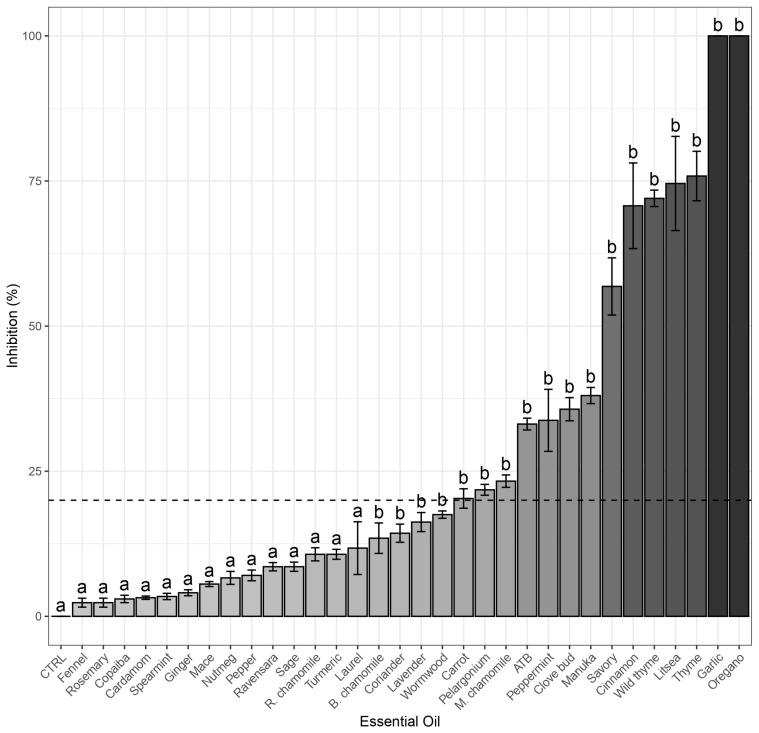
Inhibition (%) of selected essential oils compared to control against *S. aureus,* Dunnet (Control = a, *p* ≤ 0.05 = b), the dotted line indicates 20% inhibition limit. Essential oils below this limit were not tested further.

**Figure 2:**
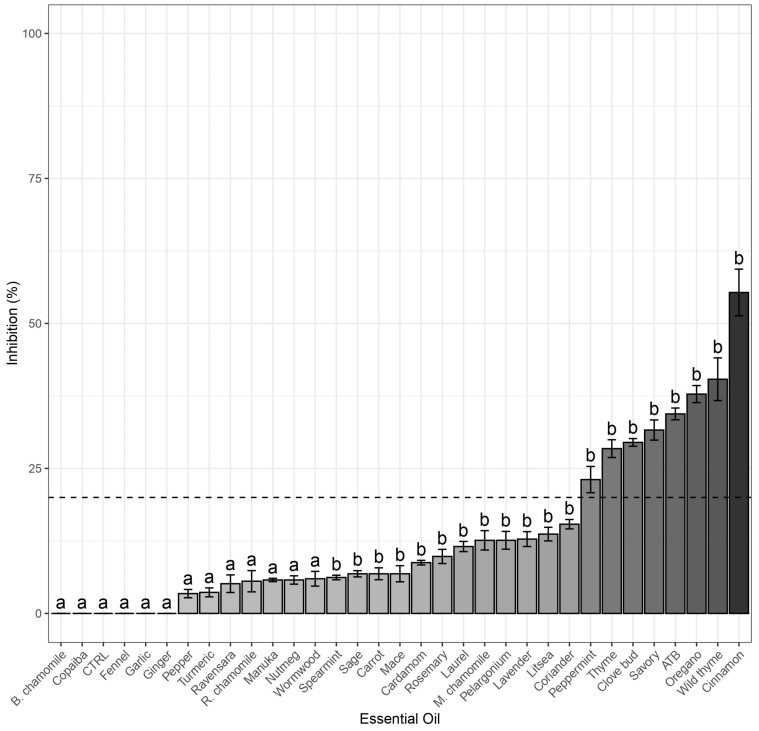
Inhibition (%) of selected essential oils compared to the control against *E. coli* and Dunnet’s test (Control = a, *p* ≤ 0.05 = b), the dotted line indicates 20% inhibition limit. Essential oils below this limit were not tested further.

All 31 EOs were tested for their antibacterial effect against *E. coli* (Gram-negative) and *S. aureus* (Gram-positive) using the agar disc diffusion method in Petri dishes. Based on the results, the most promising EOs, which demonstrated significant pathogen inhibition (inhibition zone greater than 20 mm after 24 h of cultivation), were selected for further testing. The strongest inhibition against *S. aureus* was observed in the following descending order: oregano, garlic, pelargonium, cinnamon, thyme, wild thyme, clove bud, savory, manuka, peppermint, litsea, carrot, and Moroccan chamomile (Figure 1). In the case of *E. coli*, the EOs with the highest antibacterial activity ranked as follows: oregano, thyme, wild thyme, clove bud, peppermint, cinnamon, and savory (Figure 2).

### 2.2. Determination of MIC of Selected EOs by Broth Microdilution Assay

A total of 13 EOs exhibited strong antimicrobial activity against *S. aureus*, while 7 EOs were effective against *E. coli*. These EOs were further tested in 96-well microplates to determine their MIC values for each individual EO (Table 1). For *S. aureus*, the lowest MIC values were observed for oregano and garlic EOs (MIC = 128 µg/mL), followed by pelargonium, cinnamon, thyme, and wild thyme EOs (MIC = 256 µg/mL). Moderate inhibition was observed for clove bud, savory, and manuka EOs (MIC = 512 µg/mL). Peppermint and litsea EOs showed lower effectiveness (MIC = 1024 µg/mL), while carrot and Moroccan chamomile EOs exhibited the weakest inhibition (MIC = 4096 µg/mL).

Similar results were observed for *E. coli*. The strongest inhibition was recorded for oregano EO (MIC = 128 µg/mL), followed by thyme EO (MIC = 256 µg/mL). Wild thyme, clove bud, and peppermint EOs displayed moderate inhibition (MIC = 512 µg/mL). Cinnamon and savory EOs exhibited the weakest antibacterial effects (MIC = 1024 µg/mL).

### 2.3. Interactions of EOs in Mixtures

The FIC index was calculated based on MIC values for each EO combination. Combinations showing positive interactions are listed in Table 2, along with their MIC and FIC index values. For *S. aureus*, one synergistic combination was identified: pelargonium/garlic (FIC = 0.375). Additionally, three combinations showed an additive effect: garlic/Moroccan chamomile (FIC = 0.516), Moroccan chamomile/oregano (FIC = 0.516), and carrot/clove bud (FIC = 0.562). For *E. coli*, two additive effects were observed: thyme/oregano (FIC = 0.75) and peppermint/wild thyme (FIC = 1).

### 2.4. The Composition of Selected EOs

GC-MS/MS analysis revealed the chemical composition and percentage of various compounds present in the 13 most active antibacterial EOs. EO components occurring at concentrations above 2% are listed in Table 3. Detailed chromatographic data are listed in Tables S1 and S2.

## 3. Discussion

In this study, 31 individual EOs (Table 4) were screened against *E. coli* and *S. aureus*, which are significant Gram-negative and Gram-positive human pathogenic bacteria, respectively. For all effective EOs, the minimum inhibitory concentration (MIC) was determined for both pathogens, and the fractional inhibitory concentration (FIC) index was calculated for their combinations.

Based on the screening results, the following oils were selected for further testing. Against *S. aureus*, 13 EOs showed antibacterial activity: oregano, garlic, thyme, litsea, wild thyme, cinnamon, savory, manuka, clove bud, peppermint, Moroccan chamomile, pelargonium, and carrot. Among these, oregano, garlic, thyme, litsea, wild thyme, cinnamon, savory, manuka, and clove bud exhibited stronger inhibition than the antibiotic oxytetracycline (Figure 1). Against *E. coli*, 7 EOs demonstrated significant inhibition: cinnamon, wild thyme, oregano, savory, clove bud, thyme, and peppermint. Of these, cinnamon, wild thyme, and oregano exhibited a stronger inhibition effect than oxytetracycline (Figure 2). Due to the higher antibacterial activity of some EOs compared to antibiotics, it is not surprising that EOs are often combined with antibiotics to enhance their effectiveness, particularly against resistant bacterial strains [31,32]. In general, EOs exhibited higher antimicrobial activity against *S. aureus* than *E. coli*, which aligns with the known fact that Gram-positive bacteria are more susceptible than Gram-negative bacteria [16,33] due to differences in their cell membrane composition [34]. Hydrophobic substances can more easily penetrate Gram-positive bacterial cells due to their membrane structure [14].

It has been reported that EOs have higher antimicrobial activity than their main components alone, highlighting the importance of minor components and their potential synergistic actions [7,14]. Even substances that do not exhibit antimicrobial activity individually may significantly enhance the antimicrobial effects of other compounds [35], for example, by changing the elasticity of membranes [17], inhibition of efflux pumps [18], induction of oxidative stress by combination with substances contained metal ions [36] or disruption and prevention of the biofilm [37]. In our study, the composition of the 13 most active antibacterial EOs against *E. coli* and *S. aureus* was determined (Table 3).

Oregano EO exhibited the highest antibacterial activity against both Gram-positive and Gram-negative bacteria, a finding consistent with other studies [22,38]. Among EOs with similar compositions, thyme, wild thyme, and savory also demonstrated high antimicrobial effects. These EOs have been shown to be highly effective even against methicillin-resistant *S. aureus* (MRSA) isolates [14]. Their primary active compounds are carvacrol and thymol [22,39], which aligns with our findings (Table 3). These compounds exhibit stronger antimicrobial activity than their precursor monoterpenes, *p*-cymene and γ-terpinene, and are responsible for the overall antibacterial effect of the mentioned EOs [5,40].

The concentration of the main compounds correlates with the antimicrobial activity observed. The oregano contained 73.56% carvacrol, thyme contained 46.30% thymol, wild thyme contained 16.33% thymol and 15.38% carvacrol, and savory contained 41.67% carvacrol. Wild thyme had the lowest combined concentration of these phenols (31.71%) but demonstrated better antimicrobial activity against both tested bacteria than savory, which contained a relatively high amount of carvacrol (41.67%) but no thymol (under the detection limit). This suggests that a combination of these main components likely leads to a positive interaction. A similar trend was observed in the combination of oregano (73.56% carvacrol) and thyme (46.30% thymol) EOs against *E. coli*.

The composition of thyme varies significantly and can contain different main compounds [39]. The MIC values of thyme EO from different sources also differ considerably [16]. Therefore, it is necessary to determine the composition and monitor even minor components. [40] reported an MIC of thyme against *E. coli* of 750 µg/mL, which is higher than the value found in our study (256 µg/mL). However, the thyme in their study contained only 23% thymol, whereas the thyme in our study contained twice as much (46%). Additionally, minor components such as *p*-cymene can enhance the antimicrobial effects of major compounds through synergistic interactions [41].

Pelargonium essential oil exhibited very high antibacterial activity against *S. aureus* (Table 1), which is consistent with previous findings [16,42], including activity against MRSA strains [43]. Pelargonium EO also demonstrated strong antifungal activity [44]. The main component of pelargonium EO is citronellol [41]. However, geraniol, the second most abundant component, has been shown to have even greater antimicrobial activity [45].

Cinnamon EO also exhibited a strong antimicrobial effect against *S. aureus*, with lower activity against *E. coli*, which aligns with the findings of Thielmann et al. [16]. Earlier studies suggest that the antibacterial activity of cinnamon EO is mainly due to its major component, cinnamaldehyde [46]. Cinnamon with a higher cinnamaldehyde content is potentially more effective than that with a higher eugenol content, as cinnamaldehyde has a greater antimicrobial effect [47] while also exhibiting lower toxicity to multicellular organisms [48]. El Atki et al. [31] demonstrated a significant synergistic effect of cinnamon EO with various antibiotics against both *S. aureus* and *E. coli*.

In the case of clove bud EO, an MIC value of 512 µg/mL was observed for both tested pathogens. The MIC values reported here are approximately within the midrange of those found by Thielmann et al. [16]. Similarly, Radünz et al. [49] concluded that clove bud EO exhibits the same MIC values against *E. coli* and *S. aureus*, comparable to our findings. In contrast, Prabuseenivasan et al. [46] reported that *E. coli* is more susceptible to clove bud EO than *S. aureus*. The primary compound of clove bud EO is eugenol, which is known for its antimicrobial activity against a wide range of pathogens. In addition to disrupting bacterial cell membranes, eugenol also affects bacterial enzymes [50,51]. Clove bud EO appears to exhibit stronger antimicrobial activity than eugenol alone, likely due to additive or synergistic effects among its constituents [52].

Peppermint EO is known for its significant antibacterial [38] and acaricidal effect [48]. In this study, peppermint EO exhibited greater antibacterial activity against *E. coli* than *S. aureus*, which is consistent with the findings of Thielmann et al. [16], who observed a very low MIC value for *E. coli* and a higher MIC value for *S. aureus*. However, the MIC values for *S. aureus* varied widely, likely due to differences in the composition of active compounds [10]. The most abundant components of peppermint EO were limonene, menthol, and α-pinene. Limonene and menthol have demonstrated strong acaricidal and antimicrobial effects [53], while α-pinene is known for its inhibitory activity against bacteria [54].

Other EOs with significant antibacterial effects included those from litsea, manuka, carrot, Moroccan chamomile, and garlic. However, the aforementioned EOs exhibited antimicrobial potential only against *S. aureus*. Previous studies have demonstrated a significant antimicrobial effect against Gram-positive bacteria, including MRSA [55]. The main components of litsea EO are citral A, citral B, and limonene. Liu and Yang [56] support our findings that citral is the primary active component of litsea EO and is likely responsible for its strong antimicrobial effects against Gram-positive bacteria [16].

The most abundant constituents of manuka EO are calamenene and leptospermone. Calamenene-containing EOs exhibit high antimicrobial and fungicidal activity, are effective against various pathogens (including MRSA strains), and possess strong antioxidant properties [57].

Carrot EO and chamomile EO demonstrated promising results against *S. aureus* but not against *E. coli*, which is consistent with other findings [58,59]. α-Pinene was one of the predominant components and is likely the primary contributor to the EO’s antimicrobial activity [54].

Garlic EO is widely used as an antibacterial agent against a broad spectrum of pathogens [60]. In this study, garlic EO exhibited strong antibacterial activity against *S. aureus* but not against *E. coli*. However, Seydim and Sarikus [61] reported similar antimicrobial activity against both *E. coli* and *S. aureus*. Although diallyl sulfides were identified as the main compounds in garlic EO, as described by García-Díez et al. [62], the concentration and proportions of antimicrobial compounds can vary significantly depending on the cultivar, climatic conditions, and other factors [63]. This variability is likely the main reason for inconsistencies among different studies.

In the case of FIC results, among all tested combinations, the combination of pelargonium and garlic EOs exhibited the strongest antimicrobial activity (MIC = 32 µg/mL) with a synergistic effect against *S. aureus* (FIC = 0.375). The synergistic effect of pelargonium in combination with antibiotics has also been reported [42,44,64]. Garlic also appears to have potential for synergistic or additive effects in certain combinations. Zhang and Wang [65] reported an additive effect of the garlic/citronella combination against *S. aureus*, while Ji et al. [66] observed several synergistic combinations of garlic EO against fungal pathogens. The combination of garlic and moroccan chamomile exhibited a positive interaction in the form of an additive effect against *S. aureus* (MIC = 64 µg/mL, FIC = 0.516. Moroccan chamomile and pelargonium primarily contain terpenoids such as α-pinene and 1,8-cineole, or eudesmol, linalool, and isomenthone, respectively. These substances primarily act by disrupting bacterial cell membranes, causing leakage of intracellular contents [67,68]. In contrast, garlic mainly contains organosulfur compounds such as diallyl disulfide, diallyl trisulfide, and diallyl sulfide, which inhibit the enzymatic pathways of bacteria and viruses, particularly by disrupting cysteine metabolism and reacting with thiol groups in bacterial enzymes and proteins, leading to their inactivation and the disruption of metabolic processes [69]. Thus, garlic appears to be a promising candidate for synergistic or additive effects in multiple combinations.

Another positive (additive) interaction was observed in the moroccan chamomile/oregano combination (MIC = 64 µg/mL, FIC = 0.516) against *S. aureus*. Previous studies have reported the synergistic and/or additive effects of oregano [13,22]. No data on fractional tests of moroccan chamomile were found; however, the trend of positive interactions between phenols, such as carvacrol, and less effective terpenes is well established [41]. A similar trend was observed in the clove bud/carrot combination (MIC = 256 µg/mL, FIC = 0.562), where the phenolic compound eugenol, the main component of clove bud EO, interacts with α-pinene, one of the primary components of carrot EO. Furthermore, clove bud EO and its major compound, eugenol, are known to form successful combinations with various antibiotics [50], as well as with other EOs [70].

The most effective combination against *E. coli* was thyme/oregano, with an MIC of 64 µg/mL and an FIC index of 0.75. The observed additive effect is likely due to the positive interaction between thymol and carvacrol, their primary EO constituents, which is consistent with the findings of previous studies [13,22]. The combination of thymol and carvacrol increases cell permeability, disrupts pH gradients, and causes leakage of inorganic ions [13]. Several studies have reported the synergistic effects of these compounds against other bacterial species [38,71], while others have suggested that highly antimicrobial thymol, when used in combinations, leads to positive interactions but not necessarily a clear synergistic effect [5,13,22].

The second most potent combination against *E. coli* was peppermint/wild thyme (MIC = 256 µg/mL, FIC index = 1). Peppermint has been reported to exhibit synergistic antimicrobial activity with resveratrol against *S. aureus* [72] and with the antibiotic meropenem against *E. coli* [73]. However, no evidence of a positive interaction between peppermint and wild thyme, or their major components, was found in the literature. The synergistic effect of peppermint and wild thyme is likely due to the action of peppermint terpenoids (menthol and limonene). Menthol is lipophilic and interacts with the lipid layer of bacterial membranes [74]. Wild thyme contains linalool and borneol, which can inhibit enzymes responsible for protein synthesis and metabolism of microorganisms. Borneol can generate reactive oxygen species that cause damage to cellular components such as lipids and proteins [75]. All of these compounds has a different mechanisms of action, which could explain their positive interaction.

Linalool was present in thyme (4.93%) and pelargonium (5.22%) in similar amounts, yet the combinations of peppermint EO with these EOs did not result in any positive interactions. Therefore, it is unlikely that the observed effect resulted from linalool interaction. Instead, the combination of major components such as thymol, carvacrol, *p*-cymene, limonene, and menthol appears to be more significant.

## 4. Materials and Methods

### 4.1. Bacterial Strains Used in the Study

The Gram-negative bacterium *Escherichia coli* CCM 4517 and the Gram-positive bacterium *Staphylococcus aureus* CCM 4516 were used as model organisms. The strains were obtained from the Czech Collection of Microorganisms (CCM), Brno, Czech Republic. The microbial cultures were inoculated on Mueller–(MH, Carl Roth GmbH, Karlsruhe, Germany) agar and incubated at 37 °C for 24 h. The inoculum suspension was prepared by collecting colonies from 24-h cultures and suspended in sterile 0.9% aqueous NaCl solution. The density was adjusted to match the turbidity of a 0.5 McFarland standard (10^8^ colony-forming units [CFU]/mL) using SystemSURE Plus (Hygiena, Camarillo, CA, USA) [31].

### 4.2. EOs Used in the Study

A total of 31 EOs were obtained from the company AKH a.s. (Tachov, Czech Republic) and stored at 4 °C in dark glass bottles until use. The list of EOs, including their Latin names, countries of origin, and the plant parts used, is provided in Table 4.

### 4.3. Agar Disc Diffusion Method

The screening of EOs was performed using the agar disc diffusion method according to El Atki et al. [31]. Briefly, an overnight bacterial culture grown at 37 °C was diluted in sterile 0.9% aqueous NaCl solution (Sigma-Aldrich, Schnelldorf, Germany) to a density of 0.5 McFarland (10⁸ CFU/mL), and 100 µL was spread on the surface of MH agar plates. Sterile 6 mm diameter filter discs (Whatman No.3, Cytiva, Maidstone, UK) were impregnated with 10 µL of EO per disc and placed on the inoculated MH agar plates. All tests were performed in five replicates. The antibiotic oxytetracycline was included as a positive control. The plates were incubated at 37 °C for 24 h, and the antibacterial effect was evaluated by measuring the inhibition zones. Based on the inhibition zones, inhibition as a percentage compared to the control was calculated. The percentage inhibitions are presented as mean values with a 95% confidence interval. EOs that showed an inhibition greater than 20% compared to the control in the screening test (disc diffusion test) were subjected to further evaluation using the broth microdilution assay.

### 4.4. Broth Microdilution Assay

To determine the MIC_100_ (complete inhibition), the broth microdilution assay was conducted according to Gutierrez et al. [19] with slight modifications. Given the large number of possible EO combinations, only the most effective EOs (1:1 ratio) that produced an inhibition zone greater than 20 mm were selected for further testing (78 combinations for *S. aureus* and 21 combinations for *E. coli*). The assays were performed in sterile 96-well microplates (transparent, F-bottom) (P-Lab, Prague, Czech Republic). The EO concentrations ranged from 1024 to 32 µg/mL in two-fold serial dilutions for both bacterial species. Each concentration was tested in triplicate. A total of 100 µL of 2× concentrated MH broth medium was added to each well. Then, 100 µL of EO stock solution (prepared at a concentration of 4096 µg/mL in 2× concentrated MH broth medium) was added to the first row of wells (row A). From these, 100 µL of serial dilutions were transferred to five consecutive wells (rows B–F). Rows G and H served as negative (bacteria and medium only) and sterility (medium only) controls. Each well contained 100 µL of EO solution in MH broth medium with 1% DMSO (Sigma-Aldrich, USA). Four EOs/combinations were tested in parallel on each plate. Bacterial cultures were adjusted to 0.5 McFarland in sterile 0.9% aqueous NaCl solution and further diluted 200× to obtain a final concentration of 5 × 10^5^ CFU/mL. A 100 µL bacterial suspension was added to each well (except the sterility control), resulting in a final cell count of 5 × 10^4^ CFU/well. Prior to incubation at 37 °C for 24 h, the inoculated plates were shaken orbitally for 10 s on a plate reader (BIO-RAD xMark, Hercules, CA, USA), and the optical density at 590 nm was measured. A second optical density measurement was taken after incubation, followed by another 10 s of shaking. The MIC was defined as the lowest concentration that inhibited bacterial growth after 24 h at 37 °C.

### 4.5. Positive Interactions of the Most Efficient EOs in Combinations

EO combinations were assessed for potential additive or synergistic effects using the fractional inhibitory concentration (FIC) index. The experiments were performed according to Gutierrez et al. [19] with slight modifications, as described in the broth microdilution assay section. The FIC index was calculated using the following formula:

FIC_A_ = MIC_A_ combined/MIC_A_ alone

FIC_B_ = MIC_B_ combined/MIC_B_ alone

FIC = FIC_A_ + FIC_B_

The FIC was calculated from the final MIC value of the individual EOs. The FIC_A_ and FIC_B_ values represent the fractional MICs of individual EOs in a combination that resulted in bacterial growth inhibition. The possible effects of EO combinations are summarized in Table 5.

### 4.6. Determination of MCs of the Selected EOs

To maximize accuracy and reproducibility, and due to the high complexity of the matrices, the selected essential oils were analyzed by a professional company (Joh. Vögele KG, Lauffen am Neckar, Germany) specializing in this field. EO samples were diluted 1:10,000 in hexane (PENTA, Prague, Czech Republic) and analyzed using a GC-MS/MS system consisting of a TriPlus autosampler, a Trace GC Ultra gas chromatograph equipped with a TG-5MS fused silica capillary column (30 m × 0.25 mm × 0.25 μm), and a TSQ Quantum XLS mass spectrometer (Thermo Fisher Scientific, Cleveland, OH, USA). Helium was used as the carrier gas at a flow rate of 1.0 mL/min. A 1 µL sample was injected in splitless mode at 280 °C. The oven temperature was programmed as follows: it was started at 40 °C and held for 5 min, then increased to 150 °C at a rate of 3 °C/min and held for 0.5 min. Then, it was increased to 250 °C at a rate of 10 °C/min, then increased to 290 °C at a rate of 25 °C, and finally maintained at 290 °C for 10 min. The transfer line was maintained at 250 °C, and the ion source operated at 200 °C. Total ion chromatogram (TIC) mode was performed at 70 eV ionization energy, with a mass range of 50–450 *m*/*z*. To prevent detector saturation, scanning was initiated 6 min after injection. Data was processed using Thermo Xcalibur 3.0.63 software (Thermo Fisher). Component identification was based on comparisons with the NIST Mass Spectral Search Program library v2.0f (Thermo Fisher). Quantification was performed in Q3 selected ion monitoring (SIM) mode, focusing on fragmentation ions of target compounds and using an external calibration curve. Thujone (Sigma-Aldrich, St. Louis, MO, USA) was used as both an internal and external standard.

### 4.7. Statistical Analyses

All results were analyzed using a one-way analysis of variance (ANOVA) followed by Dunnet’s test. *p* Values lower than 0.05 were considered statistically significant. All analyses were performed using the software R 4.4.0.

## 5. Conclusions

Of the 31 tested EOs, 7 exhibited strong antimicrobial effects against *E. coli* and 13 against *S. aureus*. Oregano EO demonstrated the strongest antimicrobial effect against both *S. aureus* and *E. coli*. Other EOs from the Mentheae tribe (thyme, wild thyme, savory, and peppermint) also exhibited strong antimicrobial activity against both pathogens. The antimicrobial effects of these EOs are attributed to their main components, including carvacrol, thymol, γ-terpinene, *p*-cymene, limonene, and menthol. Several EO combinations exhibited positive interactions. The most effective synergistic combination was pelargonium/garlic EO against *S. aureus*, while the thyme/oregano combination exhibited an additive effect against *E. coli*. Most of the positive interactions observed in this study have not yet been described in the literature. The results of this study contribute new insights into the use of EOs and their combinations for bacterial pathogen control. Additionally, EOs represent a cost-effective and environmentally friendly solution due to their natural origin and easily degradable residues. Furthermore, EO combinations could reduce the required application dose and concentration, making the development of bacterial resistance more difficult.

## Figures and Tables

**Table 1:** MIC of the most effective EOs against *S. aureus* and *E. coli*.

*S. aureus*		*E. coli*	
EO	MIC (µg/mL)	EO	MIC (µg/mL)
Oregano	128	Oregano	128
Garlic	128	Thyme	256
Pelargonium	256	Wild thyme	512
Cinnamon	256	Clove bud	512
Thyme	256	Peppermint	512
Wild thyme	256	Cinnamon	1024
Clove bud	512	Savory	1024
Savory	512		
Manuka	512		
Peppermint	1024		
Litsea cubeba	1024		
Carrot	4096		
Moroccan chamomile	4096		

**Table 2:** MIC and FIC index of the tested EOs in combination and their interaction effects.

*Staphylococcus aureus*			*Escherichia coli*		
EO Mixtures	MIC (µg/mL)	FIC (µg/mL)	EO Mixtures	MIC (µg/mL)	FIC (µg/mL)
Pelargonium/Garlic	32	0.375 ^syn.^	Thyme/Oregano	64	0.75 ^add.^
Garlic/Moroccan chamomile	64	0.516 ^add.^	Peppermint/Wild thyme	256	1 ^add.^
Moroccan chamomile/Oregano	64	0.516 ^add.^			
Carrot/Clove bud	256	0.562 ^add.^			

Syn.—synergistic effect; add.—additive effect.

**Table 3:** The composition of main components of EOs (components in concentration > 2%).

Essential Oil	Main Components (%)
Carrot	Carotol (30.29), α-Pinene (15.46), Sabinene (10.22), β-Caryophyllene (8.31), β-Bisabolene (5.63), β-Pinene (3.08), and Caryophyllene Oxide (2.07).
Cinnamon	Trans-cinnamaldehyde (77.69), Eugenol (7.50), Limonene (2.51), and β-Caryophyllene (2.02)
Clove Bud	Eugenol (86.63), β-Caryophyllene (10.21), and α-Humulene (2.41).
Garlic	Diallyl disulfide (34.27), Diallyl trisulfide (34.20), and Diallyl sulfide (18.89).
Litsea	Citral A (Geranial) (39.04), Citral B (Neral) (29.36), and Limonene (13.74).
Manuka	Calamenene (17.92), Leptospermone (16.02), Flaveson (5.92), α-Selinene (4.62), Cadina-1,4-diene (4.50), β-Selinene (4.42), α-Copaene (4.40), Cadina-3,5-diene (3.77), α-Cubebene (2.67), Cadina-1(6),4-diene (2.60), and β-Caryophyllene (2.03).
Moroccan chamomile	α-Pinene (20.14), Germacrene (10.23), Santolina Alcohol (7.41), (E)-β-Farnesene (6.42), 1,8-Cineol (6.22), Fenchone (5.46), Limonene (5.27), and Myrcene (4.08).
Oregano	Carvacrol (73.56), *p*-Cymene (6.97), γ-Terpinene (6.03), Myrcene (2.16), and β-Caryophyllene (2.14).
Pelargonium	Citronellol (33.53), Geraniol (15.36), Citronellyl formate (7.81), 10-epi-gamma-Eudesmol (5.37), Linalool (5.22), Isomenthone (5.16), Geranyl formate (3.29), and Menthone (2.35).
Peppermint	Limonene (38.02), Menthol (16.42), α-Pinene (15.93), β-Pinene (11.47), Menthone (5.65), and Isomenthone (2.52).
Savory	Carvacrol (41.67), γ-Terpinene (35.82), *p*-Cymene (11.73), α-Terpinene (2.41), and Myrcen (2.26).
Thyme	Thymol (46.30), *p*-Cymene (17.02), β-Caryophyllene (7.41), γ-Terpinene (5.83), Linalool (4.93), and Carvacrol (2.38).
Wild thyme	Thymol (16.33), Carvacrol (15.38), *p*-Cymene (15.01), Geraniol (10.62), γ-Terpinene (10.30), Linalool (4.94), Geranyl acetate (4.20), β-Caryophyllene (2.35), Borneol (2.33), and Terpinen-4-ol (2.13).

**Table 4:** The list of EO sources, their Latin names, countries of origin, part of the plants used, and collection voucher numbers.

Common Name	Latin Name	Origin	Part of Plant	Voucher
Black pepper	*Piper nigrum*	India	berry	2017115374
Blue chamomile	*Matricaria chamomilla*	Egypt	flower	2018108255
Carrot	*Daucus carota*	France	seeds	2019100662
Cinnamon	*Cinnamomum zeylanicum*	Sri Lanka	bark	2018112546
Clove Bud	*Eugenia caryophyllata*	Indonesia	leaves, buds, twigs	2018113001
Copaiba	*Copaifera reticulata*	Brazil	resin	2018112037
Coriander	*Coriandrum sativum*	Russia	seeds	2019109145
Fennel	*Foeniculum vulgare*	Moldova	seeds	2017107690
Garlic	*Allium sativum*	China	bulb	2017107690
Ginger	*Zingiber officinale*	Sri Lanka	rhizome	2019114043
Green cardamom	*Elettaria cardamomum*	India	seeds	2017103290
Laurel	*Laurus nobilis*	Turkey	leaves	2018113872
Lavender	*Lavandula angustifolia*	France	flowering herb	2018113954
Litsea	*Litsea cubeba*	China	fruits	2018104405
Mace	*Myristica fragrans*	Indonesia	flower	2019102915
Manuka	*Leptospermum scoparium*	New Zealand	leaves and twigs	2019113318
Moroccan chamomile	*Ormenis multicaulis*	Morocco	herb	2017114089
Nutmeg	*Myristica fragrans*	Indonesia	seeds	2017110236
Oregano	*Origanum vulgare*	Moldova	herb	2017108440
Pelargonium	*Pelargonium graveolens*	Egypt	leaves, flowers	2017108440
Peppermint	*Mentha piperita*	India	herb	2016109735
Ravensara	*Ravensara aromatica*	Madagascar	leaves and twigs	2018108724
Roman chamomile	*Anthemis nobilis*	Italy	flower	2016110437
Rosemary	*Rosmarinus officinalis*	Tunisia	herb	2017101984
Sage	*Salvia officinalis*	Romania	leaves	2018110420
Savory	*Satureja montana*	Germany	herb	2016113787
Spearmint	*Mentha spicata crispa*	USA	flowering herb	2018111114
Thyme	*Thymus vulgaris*	EU	herb	2018111114
Turmeric	*Curcuma longa*	India	root	2019111945
Wild thyme	*Thymus serpyllum*	Albania	herb	2018109107
Wormwood	*Artemisia absinthium*	USA	herb	2020101503

**Table 5:** FIC index-based type of interactions of EOs.

Obtained Effect	FIC Index
Synergy	≤0.5
Addition	0.5–1

## Data Availability

Data will be provided upon request by email.

## Supplementary Materials

The following supporting information can be downloaded at: https://www.mdpi.com/article/10.3390/molecules30091864/s1, Tables S1 and S2: Detailed chromatographic data.
