# Supplementary material for: Antibacterial Activity and Chemical Composition of Popular Plant Essential Oils and Their Positive Interactions in Combination
Source: Molecules. 2025 Apr 22;30(9):1864. doi: 10.3390/molecules30091864 (PMC12073528; doi:10.3390/molecules30091864)
Supplement: Supplementary file 1 [file molecules-30-01864-s001.zip › molecules-3548827-supplementary.pdf]

Table S1. Detailed chromatographic data (RT, Width, Area, Heights)

Essential oil	Main Compound	RT (min)	Width (min)	Area (counts*s)	Height (counts)
Carrot	α-Pinene	6.172	0.0327	1.64267e5	7.74553e4
	β-Pinene	8.355	0.0377	3.27252e4	1.33089e4
	Sabinene	8.817	0.0367	1.08566e5	4.56646e4
	β-Caryophyllene	24.818	0.0421	8.83221e4	3.42743e4
	β-Bisabolene	28.829	0.0451	5.98246e4	2.04570e4
	Caryophyllene Oxide	36.167	0.0497	2.19594e4	6.97130e3
	Carotol	37.087	0.0504	3.21746e5	1.00294e5
Cinnamon	Limonene	11.562	0.0395	3.09878e4	1.22375e4
	β-Caryophyllene	25.138	0.0459	2.48568e4	8.29824e3
	trans-Cinnamaldehyde	38.096	0.0509	9.57268e5	2.87434e5
	Eugenol	40.659	0.0406	9.24137e4	3.52093e4
Clove Bud	β-Caryophyllene	25.129	0.0451	1.34675e5	4.47889e4
	α-Humulene	27.392	0.0455	3.17912e4	1.07610e4
	Eugenol	40.655	0.0412	1.14217e6	4.14230e5
Garlic	Diallyl sulfide	9.503	0.0354	6.08978e4	2.69169e4
	Diallyl disulfide	20.857	0.0413	1.10511e5	4.11622e4
	Diallyl trisulfide	30.450	0.0466	1.10266e5	3.60963e4
Litsea	Limonene	12.402	0.0466	1.38613e5	4.54884e4
	Citral B (Neral)	28.762	0.0496	2.96145e5	9.19052e4
	Citral A (Geranial)	30.288	0.0499	3.93783e5	1.21348e5
Manuka	α-Cubebene	26.173	0.0475	2.28215e4	7.50886e3
	α-Copaene	27.135	0.0484	3.76392e4	1.20774e4
	β-Caryophyllene	28.675	0.0518	1.73605e4	5.22140e3
	Cadina-3,5-diene	29.691	0.0514	3.23105e4	9.81628e3
	Cadina-1,(6)4-diene	30.437	0.0515	2.22686e4	6.57036e3
	β-Selinene	30.914	0.0528	3.78339e4	1.05714e4
	α-Selinene	31.192	0.0585	3.95035e4	1.03507e4
	Calamenene	32.073	0.0716	1.53427e5	3.40115e4
	Cadina-1,4-diene	32.351	0.0527	3.85170e4	1.16091e4
	Flaveson	32.644	0.0574	5.09522e4	1.30969e4
	Leptospermone	35.269	0.0569	1.37106e5	3.56169e4
Moroccan chamomile	a-Pinene	6.517	0.0448	1.37639e5	4.75763e4
	Myrcene	10.784	0.0424	2.78449e4	1.00138e4
	Limonene	11.912	0.0451	3.60276e4	1.23173e4
	1,8-Cineol	12.303	0.0510	4.24982e4	1.27208e4
	Fenchone	18.926	0.0482	3.72999e4	1.20058e4
	Santolina Alcohol	19.231	0.0462	5.06462e4	1.63010e4
	(E)- β-Farnesene	27.746	0.0434	4.38327e4	1.53320e4
	Germacrene	29.094	0.0518	6.99203e4	2.10037e4
Oregano	Myrcene	9.855	0.0380	2.22247e4	9.25512e3
	γ-Terpinene	12.528	0.0388	6.19691e4	2.51110e4
	p-Cymene	13.309	0.0389	7.16984e4	2.89666e4

	β-Caryophyllene	24.415	0.0471	2.20223e4	7.32129e3
	Carvacrol	40.450	0.0369	7.56504e5	3.16645e5
Pelargonium	Menthone	20.861	0.0457	2.06444e4	6.93923e3
	Isomenthone	21.807	0.0463	4.54388e4	1.50148e4
	Linalool	23.706	0.0408	4.59797e4	1.74188e4
	Citronellyl formate	25.979	0.0428	6.87495e4	2.44711e4
	Geranyl formate	28.557	0.0449	2.89320e4	1.02592e4
	Citronellol	30.439	0.0453	2.95207e5	9.76841e4
	Geraniol	32.721	0.0450	1.35242e5	4.63908e4
	10-epi-g-Eudesmol	39.411	0.0432	4.72914e4	1.76792e4
Peppermint	α-Pinene	6.041	0.0328	1.96483e5	9.23280e4
	β-Pinene	8.229	0.0372	1.41491e5	5.84435e4
	Limonene	11.165	0.0424	4.69078e5	1.63976e5
	Menthone	20.267	0.0436	6.97530e4	2.49933e4
	Isomethone	21.183	0.0436	3.10867e4	1.08041e4
	Menthol	26.035	0.0426	2.02521e5	7.23675e4
Savory	Myrcene	10.880	0.0375	2.24652e4	9.20519e3
	α-Terpinene	11.374	0.0406	2.40304e4	9.47026e3
	γ-Terpinene	13.729	0.0441	3.56711e5	1.25714e5
	p-Cymen	14.552	0.0403	1.16767e5	4.49869e4
	Carvacrol	41.738	0.0369	4.14906e5	1.73594e5
Thyme	γ-Terpinene	12.586	0.0416	5.67535e4	2.09607e4
	p-Cymene	13.490	0.0411	1.65606e5	6.21194e4
	Linalool	23.037	0.0415	4.79924e4	1.83316e4
	β-Caryophyllene	24.414	0.0435	7.20731e4	2.51317e4
	Thymol	40.316	0.0386	4.50356e5	1.77352e5
	Carvacrol	40.867	0.0395	2.31451e4	9.15315e3
Wild thyme	γ-Terpinene	13.854	0.0420	1.15119e5	4.20064e4
	p-Cymen	14.712	0.0423	1.67834e5	6.06026e4
	Linalool	24.333	0.0421	5.51844e4	2.07280e4
	β-Caryophyllene	25.936	0.0493	2.62702e4	8.44105e3
	Terpinen-4-ol	26.126	0.0536	2.37894e4	6.82971e3
	Borneol	29.226	0.0483	2.49622e4	8.24356e3
	Geranylacetat	30.776	0.0470	4.69285e4	1.52236e4
	Geraniol	33.416	0.0442	1.18695e5	4.17119e4
	Thymol	41.369	0.0384	1.82528e5	7.50864e4
	Carvacrol	41.903	0.0373	1.71898e5	7.09369e4

Table S2. Detailed chromatographic data (Calculated RI, Reference RI, Difference, Source)

Compound	Calculated RI	Reference RI	Difference	Source
Carotol	1570	1596	26	NIST
1,8-Cineol	1026	1033	7	NIST
10-epi-gamma-Eudesmol	1571	1614	43	NIST
Borneol	1174	1166	-8	NIST
Cadina-1, 4-diene	1532	1532	0	NIST
Cadina-3,5-diene	1448	1448	0	Pherobase
Calamenene	1450	1524	74	Flavornet
Caryophyllene Oxide	1568	1582	14	NIST
Citral A	1251	1245	-6	NIST
Citral B	1217	1235	18	NIST
Citronellol	1229	1228	-1	NIST
Citronellyl formate	1276	1262	-14	PubChem
Diallyl disulfide	1077	1085	8	NIST
Diallyl sulfide	861	871	10	NIST
Diallyl trisulfide	1297	1320	23	MOLBASE
Eugenol	1384	1351	-33	NIST
Fenchone	1074	1087	13	NIST
Flavesone	1569	1546	-23	Pherobase
Geraniol	1234	1254	20	Pherobase
Geranyl acetate	1362	1385	23	NIST
Geranyl formate	1284	1298	14	NIST
Germacrene	1502	1480	-22	NIST
Isomenthone	1139	1154	15	Pherobase
Leptospermone	1592	1596	4	Pherobase
Limonene	1037	1036	-1	NIST
Linalool	1084	1099	15	NIST
Menthol	1156	1173	17	Pherobase
Menthone	1132	1154	22	Pherobase
Myrcene	990	991	1	NIST
p-Cymene	1012	1024	12	NIST
Sabinene	977	975	-2	Pherobase
Santolina Alcohol	1027	1030	3	Pherobase
Thymol	1267	1288	21	NIST
trans-Cinnamaldehyde	1293	1268	-25	NIST
α-Copaene	1376	1376	0	NIST
α-Cubebene	1351	1350	-1	NIST
α-Humulene	1391	1453	62	NIST
α-Pinene	938	939	1	NIST
α-Selinene	1464	1464	0	Pherobase

α-Terpinene	1053	1012	-41	Pherobase
β-Bisabolene	1507	1505	-2	Pherobase
β-Caryophyllene	1413	1417	4	NIST
β-Farnesene	1446	1461	15	NIST
β-Pinene	933	965	32	Pherobase
β-Selinene	1487	1492	5	Pherobase
γ-Terpinene	1051	1062	11	NIST
